# Disaster in Rio Grande do Sul, Brazil: climate crisis, Brazilian Unified National Health System response, and challenges of the new times

**DOI:** 10.1590/0102-311XEN114424

**Published:** 2025-01-13

**Authors:** Carlos Machado de Freitas, Christovam Barcellos

**Affiliations:** 1 Escola Nacional de Saúde Pública Sergio Arouca, Fundação Oswaldo Cruz, Rio de Janeiro, Brasil.; 2 Instituto de Comunicação e Informação Científica e Tecnológica em Saúde, Fundação Oswaldo Cruz, Rio de Janeiro, Brasil.

The climate emergency has reached dramatic and diverse levels over the past decades. Floods, hurricanes, droughts, heat waves, as well as other extreme events, are the most visible aspect of these changes. As is well known, all of these phenomena have always occurred and have been depicted since ancient times. What is currently new is the intensity, scale, frequency, and impact with which these phenomena occur. This is due to the amount of energy accumulating in the atmosphere and oceans, changes in wind and current circulation patterns, and the increased exposure of human populations, which now occupy areas that are more vulnerable.

The disasters that struck the State of Rio Grande do Sul, Brazil, over nine months began in September 2023, followed by more intense and widespread events at late April and early May 2024. These disasters highlight two important changes: the growing risks of extreme events and their impacts, and the responses of the Brazilian Unified National Health System (SUS, acronym in Portuguese) to these disasters.

In the 2024 disaster, on May 1st, the Governor Eduardo Leite declared a state of emergency, and by May 31, 95% of the municipalities were under state of emergency ^1^. In addition to the vast extent of the disaster, the duration of the floods reached unprecedented levels, as the Guaíba estuary remained above the alert level for over a month.

This event surpassed the 2,940 hydrological disasters (intense rains, street runoffs/flash floods/inundations, and mass movements) recorded in Rio Grande do Sul State from 1993 to 2023, as shown in Table 1 ^2^. It is the largest intensive disaster regarding territorial extent on record in the past 30 years. The health impacts also exceeded those of other major extreme climatic events in the Brazil’s recent history: there were multiple municipalities affected in the Itajaí Valley, State of Santa Catarina, in 2008, as well as the states of Pernambuco and Alagoas in 2010; and the mountainous region of the State of Rio de Janeiro in 2011 (the latter being the deadliest, with nearly 1,000 immediate deaths).

**Table 1:** Comparison of immediate human impacts of disasters in Rio Grande do Sul State, Brazil, 1993-2023 and 2024.

	Total human impacts of disasters	
	1993-2023	2024
Deaths	121	169
Homeless and displaced	650,000	663,000
Affected	8,710,000	2,350,000

Source: Brazilian Ministry of Integration and Regional Development ^2^.

Among the immediate health impacts recorded in this disaster were treatments for respiratory syndromes (accounting for 25%), gastrointestinal issues (diarrhea), skin problems (such as allergies), hypertension, diabetes, minor traumas, and suspected heart attacks ^3^. Additionally, there was a rising number of reported cases of leptospirosis, hepatitis A, acute diarrheal diseases (ADD), accidental tetanus, rabies treatments, and accidents involving venomous animals ^4^.

Regarding the health system, in the first 10 days, the Brazilian Ministry of Health estimated that at least 290 facilities, including hospitals (18 completely damaged and unable to resume services in the short term, and 75 partially operational) and emergency care units (UPA, acronym in Portuguese), were affected. Of these, 250 suffered from human resource shortages, as health workers were also among the affected population. In Canoas, one of the hardest-hit municipalities, three out of four UPA and 19 out of 27 basic health units (UBS, acronym in Portuguese) were impacted by the floods ^3^.

This disaster also marked a shift in the SUS’s response capabilities due to the speed and volume of resources and professionals involved. Box 1 provides an overview of this unprecedented mobilization effort ^2^, in which the Brazilian Ministry of Health allocated significant resources, alongside measures adopted by the Rio Grande do Sul State Health Department in the first month.

**Box 1:** Brazilian Unified National Health System (SUS) actions in response to the disaster in Rio Grande do Sul, Brazil, 2024.

	ACTIONS
Coordination	The Emergency Operation Center was set up, bringing together managers from the federal, state and municipal levels of the SUS with the aim of organizing, coordinating and controlling the employed measures (May 3rd)
Expansion of the professional contingent and flexibility	The National Force of the Brazilian Unified National Health System (FN-SUS) mobilized 60 professionals (aero-medics to hard-to-reach areas, mobile teams to work in shelters and care in field hospitals), reaching more than 300 professionals by late May
	Flexibility in the work of the 1,500 More Doctors Program professionals working in 300 municipalities in Rio Grande do Sul State. Level 1 physicians must work on call and at other levels of care, according to local needs and in municipalities other than the one in which they work, while exchange doctors must work exclusively in primary care services
	Hiring 890 more temporary professionals to work in emergencies until December 31, 2024, and opening of nine ICU beds and 100 clinical beds
Transfer of patients	Transfer of hospitalized patients in the most affected and isolated municipalities by ambulances or helicopters
Creation of temporary units to expand services	Field hospitals were set up in the municipalities of Canoas, Porto Alegre, São Leopoldo and Novo Hamburgo on May 5, 14, 18 and 25, respectively, with an average capacity of 200 patients a day each
	Sending the largest warship in Latin America to serve as a medical base for patients evacuated from hospitals in the southern part of the state, which allowed the aeromedical teams to expand their service
Medicines and supplies	Dispatch of 100 emergency kits containing 32 types of medicine and 16 types of medical supplies (gloves, syringes, bandages), totaling a potential capacity to care for 150,000 people over one month
	73,000 vials of insulin, 617,000 insulin pens and 2.8 million application needles
	8 million basic, strategic and specialized medicines and supplies
	600 doses of immunoglobulin
	200 thermal boxes and 4,800 cooling coils
	83,000 ampoules of hospital medicine for safety in intensive care for people with respiratory insufficiency
Immunizers	Sending 1.2 million doses of vaccines against tetanus, diphtheria, hepatitis A and B, whooping cough, meningitis, rotavirus, measles, mumps, rubella, rabies and animal accidents to shelters and health units in operation
Guaranteed access to diagnosis and treatment	Testing of pregnant women and people with signs of sexually transmitted infections (STI) such as HIV, syphilis and viral hepatitis, as well as access to antiretroviral treatment
	Access to treatment for people with chronic diseases such as asthma, hypertension, diabetes, hemophilia and immune disorders, with flexibility for all those who have lost their documents or prescriptions
Indigenous populations	The Secretariat for Indigenous Health worked with Civil Defense to evacuate indigenous people living in risk areas of municipalities, with 80 partially or totally isolated communities in 40 municipalities, totaling 15,000 indigenous people
	Relief actions in affected villages involving Brazilian Ministry of Health, Doctors Without Borders and UNICEF
Alerts and guidance	Warnings on risks of leptospirosis, accidents with venomous animals and human rabies
	Guidance on: leptospirosis prophylaxis for people exposed to flood waters; how to preserve insulin in the event of a power outage; keeping vaccinations up to date; prevention of respiratory syndromes in shelters; care for the emotional health of autistic people; and essential care for the population in the post-flood period
	Guidance on vaccination actions in shelters on a priority and temporary basis (influenza, COVID-19, tetanus, hepatitis A and rabies)
Capacity building	Emergency training for health professionals on diagnosis and clinical management of infectious diseases
	Training in mental health and psychosocial care as part of the care plan

ICU: intensive care unit; UNICEF: United Nations Children’s Fund.

Source: Brazilian Ministry of Health ^3^ and Rio Grande do Sul State Health Department ^4^.

The climate emergency and environmental crises have compounded and overlapped with other public health emergencies (PHE) ^5^, as well as the precarious structural conditions of municipalities and local health systems ^6^. The immediate impacts of this event are just the tip of the iceberg when considering that disasters create new risk scenarios. Its effects can be exacerbated and prolonged in the following months and years, depending on not only immediate response capacities but also integrating surveillance and health care actions. The recovery and reconstruction of the social, economic, and sanitary conditions of the affected territories and populations are required as well ^7^.

Considering territories and populations, cumulative cascade effects (direct effects contributing to secondary effects) and/or compound effects (the combination of simultaneous or successive effects involving other events) pose new challenges to public health. Rio Grande do Sul State experienced extreme weather events, including droughts in the 2021/2022 and 2022/2023 biennia, affecting 84% and 78% of the municipalities, respectively ^8^. Another disaster in September 2023, which affected over 100 municipalities, resulting in 30 deaths, 38,000 displaced or homeless people and 1.33 million affected ^2^ preceded the 2024 one. Some territories and populations were hit by both extreme events during the nine-month period. Between these events, the Rio Grande do Sul State government declared two other PHE: a dengue epidemic on March 12, with over 100 deaths by late April, and outbreaks of severe acute respiratory syndrome on May 3rd.

Part of the public, the media, decision-makers, and health professionals perceives each of these events as specific disasters and snapshot of a moment. However, Bankoff ^9^ considers that time is the essence for understanding disasters, involving history and vulnerabilities as their underlying causes. From this perspective, the timing of disasters cannot be reduced to the period of hours, days, or weeks in which they occur. In fact, it is a continuum in which social, economic, and political processes work together to either increase or reduce vulnerabilities and risks. Thus, the living and working conditions of certain territories, populations, and social groups become a constant coexistence with PHE.

The dignity of the human person is one of the cornerstones of the *Brazilian Federal Constitution* of 1988, which establishes the reduction of social and regional inequalities and the promotion of the well-being of all as objectives, as well as the prevalence of human rights as one of its principles. In this context, addressing inequalities must be a SUS commitment, but a permanent mission of the State, as vulnerable populations tend to be and more severely and repeatedly affected, creating a vicious cycle that increasingly diminishes their recovery capacities.

In addition, there are other challenges, such as the intentional or unintentional manipulation of information and the production and dissemination of fake news, which hinder the response and the proper functioning of the health system. There is a process of invisibilization of vulnerable population groups in the media, which should be priorities for action, as well as the inherent vulnerability of the health system, which operates at the limit of its capacity on a daily basis

In the current scenario, the climate emergency, disasters, and PHE are part of a broader environmental, health, social, and political crisis, demanding better organizational and response capacities from the SUS. However, the SUS has made significant progress since the disaster in the mountainous region of Rio de Janeiro, in which the *Decree n. 7,616* ^10^, of November 17, 2011, represents a milestone in such progress, since it established the criteria for declaring Public Health Emergency of National Importance (PHENI) and created the National Force of the Brazilian Unified National Health System (FN-SUS, acronym in Portuguese). However, we must go further, using systemic approaches to both dealing with each of these events and addressing the root causes and determining processes that are not only at the origin of disasters, but also in the recovery and reconstruction processes. These approaches must be integrated to result in safer and healthier territories and populations.

We already have a clear path forward when considering the legal advances made in recent years, in addition to those within the SUS itself. At late June, 2024, *Law n. 14,904* ^11^ was enacted, which established guidelines for the development of climate change adaptation plans. It highlighted the “*identification, assessment, and prioritization of measures to address recurring natural disasters and reduce the vulnerability and exposure of environmental, social, economic, and infrastructure systems in rural and urban areas, as well as the current and expected adverse effects of climate change at the local, municipal, state, regional, and national levels*” ^11^. In 2012, *Law n. 12,608* ^12^ was enacted, instituting the Brazilian National Policy for Civil Protection and Defense, which established guidelines and actions ranging from prevention to recovery processes in disaster situations. It must integrate “...*territorial planning, urban development, health, environment, climate change, water resources management, geology, infrastructure, education, science and technology, and other sectoral policies, with a view of promoting sustainable development*”. Both laws complement the 1990 SUS *Law n. 8,080* ^13^, which includes among its competencies and duties the provision of collective needs resulting from imminent danger situations, public calamities, and epidemics. They also emphasize policies involving food, housing, basic sanitation, work, income, transportation, leisure and access to essential goods and services among the determinants and health conditions, among others.

Two national plans are being developed in 2024: the Brazilian National Climate Change Adaptation Plan and the Brazilian National Civil Protection and Defense Plan. Additionally, there is an ongoing process of structuring a National Force within the Unified Social Assistance System.

While we have made progress in legal frameworks, actions, lessons learned, and plans to address these extreme events, a reactive risk management paradigm still dominates within the State sphere regarding emergencies and disasters. The focus remains on immediate responses, with little prioritization of effective actions involving prospective risk management and the coordination of sectoral public policies aimed at prevention, recovery, and sustainable and safe reconstruction. They are essential to break the vicious cycles that constitute disasters and PHE. For example, we must acknowledge the merit of the Brazilian Ministry of Health in allocating BRL 135.9 million (equivalent to USD 26.1 million in the period) for the reconstruction and strengthening of Rio Grande do Sul’s healthcare network ^14^. However, the absence of criteria and counterpart obligations tied to funding for the reconstruction of healthcare facilities and systems based on principles such as adaptation, resilience, sustainability, and safety to handle extreme events remains a significant gap. This challenge can only be met through regional collaboration between government entities to overcome the technical and financial limitations of municipalities, as well as fulfilling the permanent role of these bodies and executing long-term planning, far beyond the four-year terms of local managers. Another challenge involves the SUS and extends beyond it, requiring an integrated and systemic approach with various other sectors whose policies shape the social determinants of health at the municipal and regional levels. After all, disasters have no borders and require strong regional, intersectoral, and intergovernmental coordination to reduce the diverse processes of vulnerability in territories and populations.

We are facing new times and challenges, experiencing extreme events that result in disasters that escalate into unprecedented PHE. We need faster responses and resources to tackle these events, but also changes in risk management paradigms for extreme events and systemic public policies.
