# Supplementary material for: Disaster in Rio Grande do Sul, Brazil: climate crisis, Brazilian Unified National Health System response, and challenges of the new times
Source: Cad Saude Publica. 2025 Jan 13;40(11):e00114424. doi: 10.1590/0102-311XEN114424 (PMC11729383; doi:10.1590/0102-311XEN114424)
Supplement: Translation

# Translation: Desastre no Rio Grande do Sul, Brasil: crise climática, resposta do Sistema Único de Saúde e desafios dos novos tempos

**DOI:** 10.1590/0102-311XPT114424

**Authors:** Carlos Machado de Freitas, Christovam Barcellos

**Affiliations:** 1 Escola Nacional de Saúde Pública Sergio Arouca, Fundação Oswaldo Cruz, Rio de Janeiro, Brasil.; 2 Instituto de Comunicação e Informação Científica e Tecnológica em Saúde, Fundação Oswaldo Cruz, Rio de Janeiro, Brasil.

A emergência climática vem adquirindo graus dramáticos e diversificados ao longo das últimas décadas. A face mais visível dessas mudanças é a ocorrência de eventos extremos como inundações, furacões, secas e ondas de calor. Obviamente, todos esses fenômenos sempre ocorreram e são retratados desde a Antiguidade. O novo é a intensidade, a extensão, a frequência e o impacto, devido tanto à quantidade de energia que vem se acumulando na atmosfera e nos oceanos e às alterações de padrões de circulação de ventos e correntes, quanto à maior exposição de populações humanas que ocupam locais vulneráveis.

Os desastres que atingiram o Rio Grande do Sul, Brasil, no espaço de nove meses, primeiro em setembro de 2023 e, depois, com o de maior intensidade e extensão, entre final de abril e início de maio de 2024, marcam duas mudanças importantes, no que se relaciona aos riscos crescentes de eventos extremos e seus impactos e nas respostas do Sistema Único de Saúde (SUS) aos desastres.

No desastre de 2024, em 1º de maio, o Governador Eduardo Leite decretou estado de calamidade pública, chegando ao dia 31 de maio com 95% dos municípios nessa situação ^1^. Além da sua grande extensão, a duração desse desastre alcançou marcas raramente verificadas para inundações, tendo o nível do estuário Guaíba permanecido mais de um mês acima da cota de alerta.

Esse evento supera em números os 2.940 desastres hidrológicos (chuvas intensas, enxurradas/inundações/alagamentos e movimentos de massa) registrados no Rio Grande do Sul entre 1993 e 2023, conforme demonstrado na Tabela 1 ^2^. Trata-se do maior desastre intensivo em termos de extensão territorial registrado nos últimos 30 anos. Os impactos humanos e à saúde também superam os de outros eventos climáticos extremos que foram marcantes na história recente do país, como os que atingiram múltiplos municípios simultaneamente no Vale do Itajaí em Santa Catarina em 2008, Pernambuco e Alagoas em 2010, e Região Serrana no Rio de Janeiro em 2011 (esse foi o maior em número de óbitos imediatos, chegando a quase mil).

**Tabela 1:** Comparativo dos impactos humanos imediatos desastres no Rio Grande do Sul, Brasil, 1993-2023 e 2024.

	Total dos impactos humanos em desastres	
	1993-2023	2024
Óbitos	121	169
Desabrigados e desalojados	650.000	663.000
Afetados	8.710.000	2.350.000

Fonte: Ministério da Integração e do Desenvolvimento Regional ^2^.

Dos registros de efeitos imediatos sobre a saúde desse desastre, destacam-se os de atendimentos para síndromes respiratórias (representando 25%), gastrointestinais (diarreias), problemas de pele (como alergias), hipertensão, diabetes, pequenos traumas e suspeitas de infarto ^3^; além de um número crescente de casos notificados de leptospirose, hepatite A, doenças diarreicas agudas (DDA), tétano acidental, atendimento antirrábico e acidentes com animais peçonhentos ^4^.

Sobre o sistema de saúde, nos primeiros dez dias, o Ministério da Saúde estimou que pelo menos 290 estabelecimentos de saúde, como hospitais (18 foram totalmente danificados sem condições de voltar a realizar atendimentos no curto prazo e 75 com funcionamento parcial) e unidades de pronto atendimento (UPA), foram atingidas, sendo 250 com recursos humanos prejudicados, uma vez que os trabalhadores da saúde também estavam entre os afetados. No Município de Canoas, um dos mais impactados, 3 de 4 UPA e 19 das 27 unidades básicas de saúde (UBS) foram afetadas pelas inundações ^3^.

Esse desastre marca também uma mudança de patamar no que concerne às capacidades de resposta do SUS, pela rapidez e pelo volume de recursos e profissionais envolvidos. No Quadro 1, é apresentado um breve resumo das ações do SUS envolvendo grande volume de recursos do Ministério da Saúde, em conjunto com medidas adotadas pela Secretaria de Saúde do Rio Grande do Sul no primeiro mês, oferecendo uma ideia desse esforço inédito de mobilização ^2^.

**Quadro 1:** Ações do Sistema Único de Saúde (SUS) em resposta ao desastre do Rio Grande do Sul, Brasil, 2024.

	AÇÕES
Coordenação	Instalado o Centro de Operação de Emergência, articulando gestores das esferas federal, estadual e municipal do SUS com o objetivo de organização, coordenação e controle das medidas empregadas (3 de maio)
Ampliação do contingente de profissionais e flexibilização	Força Nacional do Sistema Único de Saúde (FNS-SUS) mobilizou 60 profissionais (aeromédicos para chegar às áreas de difícil acesso, equipes volantes para atuar em abrigos e atendimento em hospitais de campanha) chegando a mais de 300 profissionais até o final de maio
	Flexibilização de atuação dos 1,5 mil profissionais do Mais Médicos que atuam em 300 municípios do Rio Grande do Sul - os de nível 1 podendo atender em plantões e outros níveis de atenção, de acordo com as necessidades locais e em municípios diferentes do que atuam e os intercambistas exclusivamente nos serviços da atenção primária
	Contratação de mais 890 profissionais temporários para atuarem na emergência até 31 de dezembro de 2024, com abertura de nove leitos de UTI e 100 leitos clínicos
Transferência de pacientes	Transferência de pacientes internados nos municípios mais afetados e isolados por ambulâncias ou helicópteros
Criação de unidades temporárias para ampliar atendimentos	Foram instalados hospitais de campanha nos municípios de Canoas, Porto Alegre, São Leopoldo, Novo Hamburgo, respectivamente nos dias 5, 14, 18 e 25 de maio, com capacidade média de cerca de 200 atendimentos diários cada
	Envio do maior navio de guerra da América Latina para servir como base médica para pacientes evacuados de hospitais da parte sul do estado e permitindo as equipes de aeromédicos ampliarem atendimento
Medicamentos e insumos	Envio de 100 *kits* de emergência contendo 32 tipos de medicamentos e 16 tipos de insumos (luvas, seringas, ataduras), totalizando potencial de atender 150 mil pessoas durante um mês
	73 mil frascos de insulina, 617 mil canetas e 2,8 milhões de agulhas de aplicação
	8 milhões de medicamentos e insumos dos componentes básico, estratégico e especializado
	600 doses de imunoglobulina
	200 caixas térmicas e 4,8 mil bobinas de resfriamento
	83 mil ampolas de medicamentos hospitalares para a segurança na terapia intensiva de pessoas com insuficiência respiratória
Imunizantes	Envio de 1,2 milhão de doses das vacinas contra tétano, difteria, hepatites A e B, coqueluche, meningite, rotavírus, sarampo, caxumba, rubéola, raiva e acidentes com animais disponibilizados nos abrigos e unidades de saúde em funcionamento
Garantia de acesso aos diagnósticos e tratamentos	Realização de exames de gestantes e pessoas com sinais de infecções sexualmente transmissíveis (IST) como HIV, sífilis e hepatites virais e acesso a tratamento antiretroviral
	Acesso ao tratamento de pessoas com doenças crônicas como asma, hipertensão, diabetes, hemofilia e desordens imunológicas com flexibilização para todos que perderam seus documentos ou receitas.
Populações indígenas	Secretaria de Saúde Indígena articulou com Defesa Civil evacuação de indígenas residentes nas áreas de risco dos municípios, sendo 80 aldeias parcialmente ou totalmente isoladas, em 40 municípios e totalizando 15 mil indígenas
	Ações de socorro em aldeias atingidas envolvendo Ministério da Saúde, Médicos Sem Fronteiras e UNICEF
Alertas e orientações	Alertas para riscos quanto a leptospirose, acidentes com animais peçonhentos e raiva humana
	Orientações sobre profilaxia contra leptospirose para pessoas expostas à água de enchentes; sobre como preservar insulina no caso de falta de luz; manutenção de vacinações atualizadas; prevenção de síndromes respiratórias em abrigos; cuidados com a saúde emocional de autistas; cuidados essenciais à população no pós-enchente
	Orientações sobre ações de vacinação em abrigos de forma prioritária e temporária (influenza, COVID-19, tétano, hepatite A e raiva)
Capacitações	Capacitação, em regime de urgência, de profissionais de saúde sobre o diagnóstico e manejo clínico de doenças infecciosas
	Capacitação sobre cuidados em saúde mental e atenção psicossocial como parte do plano de atendimento

UNICEF: Fundo das Nações Unidas para a Infância; UTI: unidade de terapia intensiva.

Fonte: Ministério da Saúde ^3^ e Secretaria da Saúde do Rio Grande do Sul ^4^.

A emergência climática e as crises ambientais ampliadas se combinam e se sobrepõem a outras emergências em saúde pública (ESP) ^5^, bem como as condições precárias e estruturais dos municípios e dos sistemas de saúde locais ^6^. Os impactos imediatos desse evento constituem apenas a ponta de um iceberg quando consideramos que desastres produzem novos cenários de riscos, com efeitos que poderão ser agravados e prolongados nos meses e anos seguintes, dependendo não só das capacidades de respostas imediatas, mas também da estruturação de ações integradas de vigilância e cuidados da saúde com as de recuperação e reconstrução social, econômica e sanitária dos territórios e das condições de vida ^7^.

Ao mesmo tempo, quando consideramos territórios e populações, efeitos cumulativos em cascata (efeitos diretos que contribuem para efeitos secundários) e/ou compostos (combinação de efeitos simultâneos ou sucessivos envolvendo outros eventos), novos desafios são colocados para a saúde coletiva. Nos biênios 2021/2022 e 2022/2023, o Rio Grande do Sul sofreu extremos climáticos envolvendo seca/estiagem, afetando respectivamente 84% e 78% dos municípios ^8^. O desastre de 2024 foi precedido por outro em setembro de 2023, que afetou mais de 100 municípios e resultou em 30 óbitos, 38 mil pessoas entre desalojados e desabrigados e 1,33 milhões afetados ^2^. Parcelas dos territórios e das populações foram afetadas pelos dois eventos extremos no intervalo de nove meses. E, entre ambos, o governo do Rio Grande do Sul declarou outras duas situações de ESP: a epidemia de dengue em 12 de março, com mais de 100 óbitos até final de abril; e surtos de síndrome respiratória aguda grave em 3 de maio.

Na percepção de parte de público, meios de comunicação, tomadores de decisões e profissionais de saúde, cada um desses eventos é um desastre específico e retrato de um momento. Para Bankoff ^9^, o tempo constitui a essência da compreensão dos desastres, envolvendo a história e as vulnerabilidades como causas de fundo desses eventos. Nessa perspectiva, o tempo de um desastre não pode ser reduzido somente ao período (horas, dias ou semanas) no qual o evento ocorre, mas é um *continuum* em que processos sociais, econômicos e políticos atuam para aumentar ou diminuir vulnerabilidades e riscos, transformando as condições de vida e trabalho de determinados territórios, populações e grupos sociais em um permanente conviver com situações de desastres e ESP.

Na *Constituição Federal* de 1988, a dignidade da pessoa humana está entre seus fundamentos, estabelecendo a redução das desigualdades sociais e regionais e a promoção do bem de todos como objetivos e a prevalência dos direitos humanos como um de seus princípios. Nesse contexto, o combate às desigualdades deve ser um compromisso não só do SUS, mas uma missão permanente de Estado, já que as populações vulnerabilizadas tendem a ser seguidamente e mais duramente atingidas, estruturando-se um ciclo vicioso que reduz cada vez mais suas capacidades de recuperação.

Somam-se, ainda, o enfrentamento conjunto de outros desafios, como a manipulação, proposital ou não, de informações e a produção e divulgação de *fake news*, que prejudicam o enfrentamento desses eventos e o funcionamento pleno do sistema de saúde; o processo de invisibilização nos meios de comunicação de grupos populacionais mais vulneráveis que deveriam constituir prioridades de ação; e a própria vulnerabilidade do sistema de saúde, que vive o seu cotidiano no limite operacional.

No cenário atual, a emergência climática, os desastres e as ESP integram uma crise ambiental, sanitária, social e política mais ampla, exigindo melhores capacidades de organização e resposta do SUS, que vem avançando bastante desde o desastre da Região Serrana, sendo um marco nesse processo o *Decreto nº 7.616* ^10^, de 17 de novembro de 2011, que estabeleceu os critérios de declaração de Emergência em Saúde Pública de Importância Nacional (ESPIN) e instituiu a Força Nacional do Sistema Único de Saúde (FN-SUS). Porém, é necessário ir além, com abordagens sistêmicas, tanto para a resposta a cada um desses eventos como para o enfrentamento das causas de fundo e processos de determinação que estão na sua origem dos desastres e nos processos de recuperação e reconstrução que devem ser integrados e resultar em territórios e populações mais seguros e saudáveis.

Já temos um caminho aberto, se considerarmos os avanços no marco legal ao longo dos últimos anos que se somam aos do próprio SUS. No final de junho desse ano, foi sancionada a *Lei nº 14.904* ^11^, que estabelece diretrizes para a elaboração de planos de adaptação à mudança do clima, destacando-se a “*a identificação, a avaliação e a priorização de medidas para enfrentar os desastres naturais recorrentes e diminuir a vulnerabilidade e a exposição dos sistemas ambiental, social, econômico e de infraestrutura, em áreas rurais e urbanas, bem como os efeitos adversos atuais e esperados das mudanças do clima nos âmbitos local, municipal, estadual, regional e nacional*”. Em 2012, tivemos sancionada a *Lei nº 12.608* ^12^, instituindo a Política Nacional de Proteção e Defesa Civil, estabelecendo como diretrizes envolver ações que vão desde a prevenção até os processos de recuperação em situações de desastres e devendo se integrar “...*às políticas de ordenamento territorial, desenvolvimento urbano, saúde, meio ambiente, mudanças climáticas, gestão de recursos hídricos, geologia, infraestrutura, educação, ciência e tecnologia e às demais políticas setoriais, tendo em vista a promoção do desenvolvimento sustentável*”. Ambas se somam à *Lei nº 8.080* do SUS de 1990 ^13^, que tem entre suas competências e atribuições o atendimento de necessidades coletivas decorrentes de situações de perigo iminente, calamidade pública e epidemias, ao mesmo tempo em que destaca entre os determinantes e condicionantes da saúde as políticas envolvendo alimentação, moradia, saneamento básico, trabalho, renda, transporte, lazer e o acesso aos bens e serviços essenciais, entre outros.

No ano de 2024, temos dois planos nacionais sendo elaborados - o Plano Nacional de Adaptação à Mudança do Clima e o Plano Nacional de Proteção e Defesa Civil -, além de um processo de estruturação de uma Força Nacional no Sistema Único de Assistência Social.

Se temos avançado em marcos legais, ações, aprendizados e planos para o enfrentamento desses eventos extremos, é ainda hegemônico, no âmbito do Estado, um paradigma de gestão reativa aos riscos provocados pelas emergências e desastres, com foco nas respostas imediatas e pouca priorização efetiva das ações que envolvem uma gestão de riscos prospectiva e a articulação de políticas públicas setoriais com foco nos processos de prevenção, recuperação e reconstrução de modo sustentável e seguro, que são fundamentais para romper os ciclos viciosos que constituem os desastres e ESP. Por exemplo, se devemos reconhecer o mérito da disponibilização de R$ 135,90 milhões pelo Ministério da Saúde para a reconstrução e o fortalecimento da rede de saúde gaúcha ^14^, a ausência de critérios e contrapartidas no financiamento para reconstrução de estabelecimentos e sistemas de saúde baseados nos princípios de adaptação, resiliência, sustentabilidade e segurança para lidar com eventos extremos é ainda uma grande lacuna. Esse desafio somente será enfrentado por meio de uma articulação regional entre entidades governamentais, que superam as limitações técnicas e financeiras de municípios, bem como a atuação permanente desses órgãos e o planejamento a longo prazo, muito além dos mandatos de quatro anos de gestores locais. Outro desafio envolve o SUS e vai além do mesmo, demandando uma atuação integrada e sistêmica com os vários outros setores que em suas políticas conformam os determinantes e os condicionantes da saúde no nível dos municípios e das regiões, pois os desastres não possuem fronteiras, exigindo forte articulação regional, intersetorial e interfederativa para a redução dos diversos processos de vulnerabilização no nível dos territórios e populações.

Estamos diante de novos tempos e desafios, vivenciando eventos extremos, que resultam em desastres e se exacerbam em ESP sem precedentes. Precisamos de maior rapidez nas respostas e de recursos para enfrentar esses eventos, além de mudanças nos paradigmas de gestão de riscos de eventos extremos e de políticas públicas sistêmicas.
